# Heart Failure Pharmacological Management: Gaps and Current Perspectives

**DOI:** 10.3390/jcm12031020

**Published:** 2023-01-28

**Authors:** Paolo Severino, Andrea D’Amato, Silvia Prosperi, Vincenzo Myftari, Elena Sofia Canuti, Aurora Labbro Francia, Claudia Cestiè, Viviana Maestrini, Carlo Lavalle, Roberto Badagliacca, Massimo Mancone, Carmine Dario Vizza, Francesco Fedele

**Affiliations:** Department of Clinical, Internal, Anesthesiology and Cardiovascular Sciences, Sapienza University of Rome, Viale del Policlinico, 155, 00161 Rome, Italy

**Keywords:** heart failure, acute heart failure, chronic heart failure, left ventricular ejection fraction, management, therapy

## Abstract

Proper therapeutic management of patients with heart failure (HF) is a major challenge for cardiologists. Current guidelines indicate to start therapy with angiotensin converting enzyme inhibitors/angiotensin receptor neprilysin inhibitors (ACEi/ARNI), beta blockers (BB), mineralocorticoid receptor antagonists (MRAs) and sodium glucose cotransporter 2 inhibitors (SGLT2i) to reduce the risk of death and hospitalization due to HF. However, certain aspects still need to be defined. Current guidelines propose therapeutic algorithms based on left ventricular ejection fraction values and clinical presentations. However, these last do not always reflect the precise hemodynamic status of patients and pathophysiological mechanisms involved, particularly in the acute setting. Even in the field of chronic management there are still some critical points to discuss. The guidelines do not specify which of the four pillar drugs to start first, nor at what dosage. Some authors suggest starting with SGLT2i and BB, others with ACEi or ARNI, while one of the most recent approach proposes to start with all four drugs together at low doses. The aim of this review is to revise current gaps and perspectives regarding pharmacological therapy management in HF patients, in both the acute and chronic phase.

## 1. Introduction

Proper therapeutic management of patients with heart failure (HF) is a major challenge for cardiologists [1,2,3,4]. The complexity of this multifaceted syndrome along with the increasing availability of different pharmacological weapons requires standardized approaches to maximize the impact of HF therapy on mortality and rehospitalization. 

Current European Society of Cardiology (ESC) and American College of Cardiology/American Heart Association (ACC/AHA) guidelines [1,2] indicate starting therapy with angiotensin converting enzyme inhibitors/angiotensin receptor neprilysin inhibitors (ACEi/ARNI), beta blockers (BB), mineralocorticoid receptor antagonists (MRAs) and sodium glucose cotransporter 2 inhibitors (SGLT2i) to reduce the risk of death and hospitalization due to HF, in patients with HF with reduced ejection fraction (HFrEF). 

**Figure 1:**
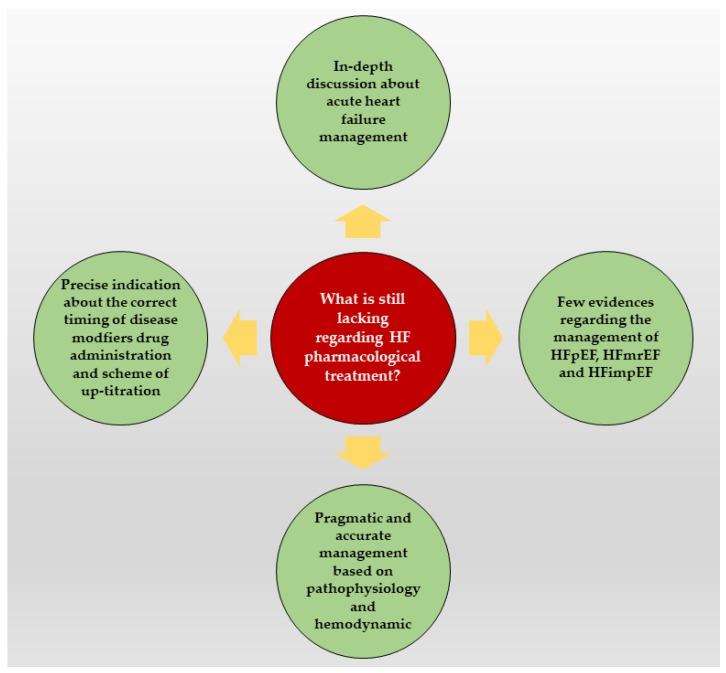
Main current gaps in heart failure pharmacological treatment. HF: heart failure; HFmrEF: heart failure with mildly reduced ejection fraction; HFpEF: heart failure with preserved ejection fraction; HFimpEF: heart failure with improved ejection fraction.

However, regarding therapeutic management of HF patients, certain aspects still need to be defined:

(i) left ventricular ejection fraction (LVEF) continues to represent the fundamental parameter for the diagnosis of HF patients, risk stratification and therapeutic management, despite its well-known limitations. Even if both European and American guidelines [1,2] proposed a LVEF-based HF classification, defining HFrEF as LVEF ≤ 40%, HF with mildly reduced EF (HFmrEF) as LVEF between 41% and 49%, and HF with preserved EF (HFpEF) as LVEF ≥ 50%, LVEF cut-offs used for the classification have varied in the guidelines over the years and a definition of a normal range is still lacking. The quantitative calculation of LVEF, defined as LV stroke volume divided by end-diastolic volume, cannot fully categorize the different types of HF patients, who often share similar clinical and prognostic characteristics and would require the same management, regardless of this echocardiographic parameter; for instance, various trials aimed to show the beneficial effects, in HFpEF patients, of the use of the main drugs already approved for HFrEF therapy, such as the Aldosterone Antagonist Therapy for Adults With Heart Failure and Preserved Systolic Function (TOPCAT) trial [5]. It evaluated Spironolactone vs. placebo and did not prove a significant reduction of primary endpoint, composed of cardiovascular death and hospitalization for HF. In addition, in the Efficacy and Safety of LCZ696 Compared to Valsartan, on Morbidity and Mortality in Heart Failure Patients With Preserved Ejection Fraction (PARAGON-HF) trial [6], the use of ARNI in HFpEF was demonstrated to improve symptoms and to reduce HF hospitalization, without significant reduction in mortality rate. Another example is the EMPagliflozin outcomE tRial in Patients With chrOnic heaRt Failure With Preserved Ejection Fraction (EMPEROR-PRESERVED) trial [7], in which Empaglifozin was shown to significantly reduce mortality and hospitalization due to HF. These studies emphasized how HF patients should be considered mostly on their common underlying pathophysiological mechanisms, rather than the pure LVEF value. The guidelines [1,2] emphasize a therapeutic management guided by LVEF and patient’s clinical profile [8]. However, both approaches have some limitations [9,10,11]. An approach based only on clinical profile can sometimes be misleading and simplistic, particularly in specific settings. Indeed, different pathophysiological mechanisms may contribute to the genesis of clinically similar scenarios, but they need to be treated differently depending on the underlying cause [12,13,14,15]. In this regard, a pragmatic approach based on pathophysiology and hemodynamic profile [14,15] may be more appropriate, particularly in the management of the acute setting; (ii) most of the proposed and discussed approaches for managing HF therapy focus on the chronic stable phase, neglecting episodes of acute decompensation; (iii) precise indication regarding the timing and sequences of drug administration, as well as the titration strategy, is lacking, both in acute and chronic settings; (iv) little evidence is provided regarding the therapeutic management of patients with HFmrEF, HFpEF and HF with improved EF (HFimpEF) because those patients are often excluded from major randomized clinical trials, despite how they may represent a large proportion of HF patients. These still unclear points are summarized in Figure 1.

The aim of this review is to revise current gaps and perspectives regarding pharmacological therapy management in HF patients, in both the acute and chronic phase.

## 2. Acute Heart Failure Management: Is a Change in the Approach Needed?

The pharmacological management of acute heart failure (AHF) is often neglected by current approaches proposed, which focus only on the chronic stable phase of HF. However, AHF is often an important part of the HF pathway because it may begin with an AHF episode, and rehospitalization due to AHF represents a recurrent event in the natural history of HF syndrome, showing a great impact on mortality and life quality [1,2,3,4]. In addition, only a proper management of the acute phase allows rapid introduction and up-titration of drugs modifying HF disease and, as suggested by the guidelines [1,2], reducing the mortality and HF rehospitalization rates [1,2]. For what concerns the therapeutic strategies in AHF setting, current guidelines [1,2] propose different algorithms based on the four clinical presentations: acute decompensated heart failure, acute pulmonary oedema, isolated right ventricular failure and cardiogenic shock (Table 1). In this scenario, drugs such as diuretics, inotropes and vasopressors are administered according to the prevailing symptoms (i.e., fluid overload, hypotension and acute respiratory failure) and clinical presentation. 

However, clinical phenotypes do not always reflect the precise hemodynamic status of patients. Furthermore, in the acute setting, LVEF evaluation alone may be misleading because it is dependent on fluid load condition and it does not consider the pathophysiological bases of the acute decompensation. To overcome these limitations, a more pathophysiological, as well as the evaluation of hemodynamic parameters may guide therapeutic choices. Stroke volume and stroke volume variation are useful to evaluate cardiomyocytes’ recruitment according to fluid filling and the Frank–Starling mechanism. For this reason, a hypotensive patient with preserved stroke volume variation may respond to fluid filling. In this regard, it is important to specify that this condition of fluid responsiveness may be quite different in patients with HF. In fact, rapid fluid filling may increase stroke volume without association with hemodynamic improvement and early decongestion, or it may further worsen HF patients’ hemodynamic. The absolute and indexed values of systemic vascular resistance (SVR) reflect the compensatory hyperactivation of the sympathetic system and, in cases of hemodynamic instability, SVR is increased due to reduced myocardial contractility and compensatory vasoconstriction. Patients with AHF and increased SVR may benefit from inodilator administration, while norepinephrine can be harmful, worsening peripheral vasoconstriction and cardiac afterload. Cardiac output and, in particular, cardiac power output (CPO) may help clinicians to evaluate responses to therapy in the acute phases, helping to decide how to manage inotropes, when to evaluate a mechanical circulatory support or consider palliative care [16]. According to the SHOCK trial registry, CPO, defined by mean arterial pressure x cardiac output/451, represents the strongest independent hemodynamic parameter of cardiogenic-shock-related mortality in the in-hospital setting [17]. Burstein et al. evaluated the applicability and the role of CPO measured through echocardiography, finding out that non-invasive CPO was inversely related to in-hospital mortality in cardiac intensive care unit (ICU) patients and that it represented an adjunctive, prognostic parameter to stratify critical cardiac ICU patients [18]. Yildiz et al. demonstrated that patients with advanced heart failure, who showed lower CPO at rest, were more prone to adverse events. CPO may indeed also be considered a valid prognostic parameter for risk stratification in advanced heart failure [19]. Furthermore, CPO showed a prognostic role in HFpEF patients in terms of adverse outcomes prediction, while other parameters of cardiac performance were not associated with HFpEF patients’ prognosis [20].

The main studies [21,22,23,24,25,26] evaluating approach based on volume status for the management of AHF and chronic HF are summarized in Table 2.

Mostly, AHF episodes are characterized by a state of fluid overload and a treatment based on diuretics and vasodilators, and oxygen and/or non-invasive ventilation is enough to stabilize patients. However, hypotension and/or end-organ hypoperfusion may also occur and, in this case, the use of inotropic and vasopressor agents may be evaluated according to the guidelines’ indications [1,2]. Even if it has been shown that the use of inotropes may have a negative effect on survival, mainly due to higher oxygen consumption and arrhythmic burden, they may contribute to restoring an adequate cardiac output, improving organ perfusion. There are three classes of inotropes that may be used, namely beta-adrenergic agonists, phosphodiesterase-3 inhibitors (PDE3i) and the calcium sensitizer or inodilators. There is a heated debate regarding the type of inotrope to be used and the results of randomized controlled trials are often conflicting or inconclusive. 

The choice of inotropic agent has to take into account the patient’s hemodynamic and pathophysiological profile. For example, in ischemic decompensated HF patients, PDE3i Milrinone shows deleterious effects [27], making either Dobutamine or Levosimendan preferable. On the other hand, PDE3i Milrinone and Levosimendan are preferred in right ventricular HF and pulmonary hypertension since they exert a vasodilatory effect on pulmonary circulation [28]. 

Levosimendan has peculiar pharmacodynamic effects. It improves the calcium sensitization of Troponin C without increasing intracellular calcium concentration. It induces vasodilation and diastolic function improvement through its activity as PDE3i, as well as adenosine triphosphate potassium (K-ATP) channels activation. Given the peculiar pharmacokinetic and pharmacodynamic features, Levosimendan is particularly useful to restore short- and medium-term hemodynamic balance in patients with acute decompensated HF due to the effects of its metabolites, which may persist up to seven days. Several trials demonstrated the rationale of Levosimendan use in the pathophysiology and hemodynamics of HF. In a sub-analysis of the Hemodynamic Evaluation of Levosimendan in Patients With pulmonary hypertension-HFpEF (HELP) trial, Brener et al. [29] demonstrated that the hemodynamic effects of Levosimendan are particularly mediated by venodilation, which reduces myocardial filling pressure determining also beneficial effects on glomerular filtration [30]. Furthermore, Levosimendan reduces pulmonary capillary wedge pressure (PCWP) and central venous pressure [29,31]. In addition, its use is preferred in patients already treated with BB because its mechanism of action is independent of the adrenoreceptors. This can be one of the causes contributing to lower mortality in patients treated with Levosimendan compared with patients treated with Dobutamine in a Survival of Patients With Acute Heart Failure in Need of Intravenous Inotropic Support (SURVIVE) trial sub-analysis [32]. Moreover, Levosimendan, particularly when administered during AHF, may reduce hospitalization length, impacting also on costs [33]. 

HF patients, particularly those with recurrent acute decompensation episodes due to a labile hemodynamic balance and residual congestion at discharge, do not tolerate guideline-directed medical therapy (GDMT). In this scenario, intermittent Levosimendan administration has demonstrated to facilitate GDMT optimization [34]. This interesting result may be justified considering the pleiotropic hemodynamic effect of Levosimendan. In fact, several studies have underlined the role of Levosimendan in stable advanced chronic HF and its impact on hemodynamic parameters stabilization. Najjar et al. demonstrated [28] a positive hemodynamic effect through the increase of cardiac output, a reduction in peripheral vascular resistance and myocardial afterload, as well as circulating N-terminal pro B-type natriuretic peptide (NT pro BNP) induced by Levosimendan [35]. However, despite positive hemodynamic effect, the use of repetitive infusion of Levosimendan in patients with chronic advanced HF provided contrasting results [36,37]. The efficacy of Levosimendan may therefore depend on the choice of the right administration timing, right hemodynamic profile and disease stage [38].

The use of diuretics is a cornerstone of AHF treatment. Despite loop diuretics, in particular Furosemide, representing the most widely used diuretics to treat congestion, several recent trials investigated the role of other diuretics in the acute setting. 

The Acetazolamide in Decompensated heart failure with Volume Overload (ADVOR) trial [39] enrolled patients with AHF and fluid overload across the spectrum of different LEVF values. It demonstrated that the addition of Acetazolamide, a diuretic acting on proximal tubule, to a loop diuretic was associated with improved diuretic response and greater successful decongestion incidence, regardless of LVEF [40,41]. The Safety and Efficacy of the Combination of Loop with Thiazide-type Diuretics in Patients with Decompensated Heart Failure (CLOROTIC) trial [42] demonstrated that the addition of hydrochlorothiazide to loop diuretic in AHF patients improved the diuretic response, without differences in terms of rehospitalization and mortality. However, treatment with hydrochlorothiazide was associated with a significant renal impairment without significant potassium imbalances [43]. 

The early administration of GDMT in patients hospitalized for AHF was reported by several trials. In particular, the effects of SGLT2i in the acute setting were investigated by the Study to Test the Effect of Empagliflozin in Patients Who Are in Hospital for Acute Heart Failure (EMPULSE) trial [44] and Efficacy and Safety of Dapagliflozin in Acute Heart Failure (DICTATE-AHF) [45]. The EMPULSE trial demonstrated that the early initiation of Empagliflozin during hospitalization was associated with early and prolonged decongestion, as well as clinical improvement [46]. The latter was associated with early and durable improvement of life quality, symptoms and physical limitation [47]. 

Another sub-analysis of the same trial showed that Empagliflozin was effective independently by baseline renal function. Moreover, early initiation of Empagliflozin was associated with an initial mild renal worsening with consequent recovery of renal function and no differences in terms of renal adverse events, compared with patients treated with placebo [48]. 

## 3. Chronic Heart Failure and Current Management Approaches: Is There a Head Combination or Are They All the Same?

According to the current guidelines [1,2], ACEi/ARNI, BB, MRAs and SGLT2i have all been proven to reduce mortality and the risk of hospitalization due to HF for all patients with HFrEF [49] (Table 1). However, the issue is still open regarding the initiation timing, as well as sequencing and up-titration strategies. For instance, it is explicitly advised to start the treatment with SGLT2i in all those patients who are already treated with the former cornerstone therapies, despite growing evidence showing the beneficial role of SGLT2i regardless of the other treatment. Furthermore, in most of the trials that have evaluated the efficacy of each molecule, it was not required for the patients to be neither on optimal GDMT nor at the up-titrated dose. Indeed, among the most reputed trials about SGLT2i, only a minority of patients, 19.4% in the Empagliflozin Outcome Trial in Patients With Chronic Heart Failure With Reduced Ejection Fraction (EMPEROR-Reduced) trial [50] and 10.4% in the Study to Evaluate the Effect of Dapagliflozin on the Incidence of Worsening Heart Failure or Cardiovascular Death in Patients With Chronic Heart Failure (DAPA-HF) trial [51], were already treated with ARNI. This is a clear example of how the timing for an optimized, safe and effective treatment strategy needs more evidence in order to be systematized. On this subject, in support of an early use of SGLT2i, both the DAPA-HF and EMPEROR-Reduced trials showed significant improvement of symptoms and reduction of cardiovascular death after, respectively, 28 and 12 days from the randomization of mainly ambulatory HF patients treated with SGLT2i, regardless of concomitant other HF therapy. This aspect is crucial knowing that the first period after discharge, the vulnerable phase, is particularly critical for HF, in terms of acute decompensation episodes [52,53]. SGLT2i use is also associated with significant improvement of life quality, assessed by the Kansas City Cardiomyopathy Questionnaire (KCCQ-12), in HFrEF patients [54]. The impact of SGLT2i on main outcomes in HF is mediated by the pleiotropic mechanisms of this class of drugs. The mechanisms responsible for cardiovascular system benefits are not completely understood and several hypotheses have been postulated, such as blood pressure control and diuretic effect. However, the main mechanism may be the switch in myocardial fuel utilization away from glucose towards consumption of fatty acids and ketone bodies [55]. Santos-Gallego et al. [56] demonstrated that the myocardium metabolic switch induced by Empagliflozin was associated with increased levels of ATP and myocardial work efficiency. These mechanisms were associated with reduced LV adverse remodelling and improved LV systolic function. 

Another gap concerns the possibility to begin ARNI in patients who are not already treated with ACEi. In the current Guidelines [1], ARNI are recommended as a replacement for an ACEi in patients with HFrEF to reduce the risk of HF hospitalization and death and may be considered in ACEi naive patients. In this regard, ACC/AHA guidelines [2] indicate to directly start ARNI, also in patients with de novo HF. Regarding the in-hospital setting, two studies have shown that ARNI are a safe alternative to ACEi. The Comparison of Sacubitril/Valsartan Versus Enalapril on Effect on NT-proBNP in Patients Stabilized From an Acute Heart Failure Episode (PIONEER-HF) trial [57] showed a significant reduction of NT pro BNP already in the first ten days in patients treated with ARNI rather than ACEi. Moreover, the rates of renal disfunction, hyperkalemia, symptomatic hypotension and angioedema did not differ between the two groups [57]. In a subgroup analysis of the Comparison of Pre- and Post-discharge Initiation of LCZ696 Therapy in HFrEF Patients After an Acute Decompensation Event (TRANSITION) trial [58], first diagnosed patients and patients with a subsequent episode of decompensated HF were randomized to initiate ARNI. Patients with first episode of acute decompensated HF treated with ARNI showed faster and greater decreases in NT pro BNP and high-sensitivity troponin-T, lower rates of HF and all-cause rehospitalization, and a higher proportion of patients achieved the therapeutic targeted dose [58]. Oh et al. [59] designed a very specific trial aiming to investigate the benefits of the early initiation of ARNI in newly diagnosed HF patients. It turned out that the subgroup who received upfront treatment with ARNI had lower rates of cardiac death and HF hospitalization. These findings are consistent with the hypothesis that an early initiation of ARNI is not only safe, but also advisable [59].

Based on the abovementioned pitfalls in the current therapeutic management of HFrEF, several alternative schemes have been proposed (Table 3). McMurray et al. [60] suggested initiating BB and SGLT2i upfront, followed by ARNI, within two weeks, and MRAs two more weeks later. This arises from the consideration that, since each drug exerts a beneficial effect of its own, the priority is to administer all of the molecules in the shortest time possible, regardless of their optimal up-titration. Moreover, since much of the benefits of foundational treatments are seen within 30 days after the treatment initiation, it is important to achieve GDMT within 4 weeks. The authors, however, underline that the proposed algorithm is most appropriate for outpatients, and more precaution is necessary in hospitalized patients [60].

Miller et al. [61] suggested a more phenotype-based approach dividing the HF drugs into three different clusters associated with three groups of symptoms: Cluster A made by SGLT2i and diuretics for volume overload, Cluster B by ARNI/MRAs for hypertension and Cluster C by BB and sinus node inhibitors for high heart rate. They advised to start the treatment according to the most prevalent clinical scenario, achieving the GDMT within 6 weeks regardless of the optimal titration [61]. 

Greene et al. [62] supported a nearly simultaneous introduction of low doses of each of the four classes of drugs, within the first week, and subsequent rapid up-titration in the following month.

What until now had only been suggested by clinical experience, is now supported by a strong piece of evidence provided by the recent Safety, Tolerability and Efficacy of Rapid Optimization, Helped by NT-pro BNP testing of Heart Failure Therapies (STRONG-HF) trial [63]. This multicenter prospective randomized study was the first to compare an upfront treatment protocol versus usual care in 1078 patients admitted for heart failure treated with suboptimal GDMT. The study ended early because of greater than expected differences in the outcomes of reduction in blood pressure levels, heart and respiratory frequency, NYHA class and NT pro BNP levels. However, in order to achieve fast optimal treatment, it was required that patients undergo a close follow-up, mainly to deal with minor side effects such as hypotension and hyperkalemia, with more visits than those following routine treatment, implying that an important effort should be made by HF centers in doubling the volume of outpatient visits. This study [63] provides robust evidence of the beneficial role and safety of a more aggressive treatment protocol, suggesting that any delays in reaching full GDMT is equivalent to denying the patient a possibility of improving their health. 

One of the most important limits to GDMT up-titration is the presence of chronic kidney disease (CKD). However, most of HF disease-modifying drugs show a nephroprotective role. CKD is a common comorbidity in HF patients, leading to higher rates of hyperkalemia, especially when combined with Renin-angiotensin-aldosterone system inhibitors (RAASi) and MRAs intake. Hyperkalemia, defined by plasmatic potassium levels higher than 5.5 mmol/L, has several negative consequences, such as frequent rehospitalization, higher rate of arrhythmias, progression to CKD and greater risk of all-cause mortality [64,65]. However, the recent possibility to use potassium binders such as Patiromer and Sodium Zirconium Cyclosilicatum (SZC) allows for the up-titration of RAASi and MRAs, despite the presence of severe and/or advanced renal failure, as well as hyperkalemia. Potassium binders guarantee the reduction of mortality and HF-related hospitalization risk also in this frail population, for which the therapeutic possibilities were scarce in the recent past. The Study to Investigate the Safety and Efficacy of ZSC in Patients With Hyperkalemia (HARMONIZE GL) trial [66], in fact, assessed the efficacy of SZC in guaranteeing normokalemia with an overall good tolerance and a low rate of treatment discontinuation due to its minor side effects, such as oedema and constipation. Additionally, the Patiromer for the Management of Hyperkalemia in Subjects Receiving RAASi Medications for the Treatment of Heart Failure (DIAMOND) trial [67] underlined the role of the potassium binder Patiromer for the optimization of therapy in patients with HFrEF. In fact, the use of Patiromer in HFrEF patients with RAASi-associated hyperkalemia was associated with a reduction in hyperkaliemia episodes and better control of plasmatic potassium values. Moreover, the use of Patiromer allowed an increased use of RAASi and MRAs doses [68]. In the recent guidelines [1,2], it is stated that potassium binders may be used in patients with chronic or recurrent hyperakalemia as soon as plasmatic potassium levels are found to be > 5.0 mEq/L, not preventing the clinician from using RAASi and MRAs even in those patients more at risk of hyperkalemia. 

Finally, a mention of another critical point, which although often underestimated, has an important impact on the success of medical therapy: the patient’s compliance. The clinician does not have to forget that a typical HF patient has to take a minimum of four pills for a single disease, often associated with other secondary drugs such as diuretics, antiplatelets and anticoagulants, or drugs for other pathologies. Not having a standardized therapeutical model can be confusing and contribute to poor patient compliance. Guidelines [1,2] stress the concept of a strict monitoring of HF patients through follow-up visits with the aim of maintaining high compliance. However, here too the question of the lack of precise periodization for follow-up visits remains open, further contributing to creating gaps and mismanagement in the already extremely complex therapy of HF. 

Beyond the role of pharmacological treatment to reduce the risk of mortality and hospitalization in HF patients, increasing evidence supports the role of several devices for HF management. Some of these devices, through accurate and invasive monitoring, identify the early phases of acute decompensation episodes, allowing an early treatment modification and reducing the risk of rehospitalization due to HF. Other devices are now recognized as adjunctive therapy for patients who are still symptomatic despite optimized medical therapy. What is more, patients with higher pulmonary artery pressure (PAP) are more at risk of HF hospitalization and mortality [69], implying that HF care also needs to be “hemodynamically guided”. Implantable hemodynamic monitoring devices, such as the CardioMEMS HF system, provide frequent PAP measurements and early detection of hemodynamic congestion by sensing changes in filling pressures, even when patients are still asymptomatic. The CardioMEMS Heart Sensor Allows Monitoring of Pressure to Improve Outcomes in NYHA Class III Heart Failure Patients (CHAMPION) trial [70] showed that management using PAP information, enabling the clinician to promptly make tailored therapeutic changes, reduced HF hospital admission by 33% during 18 months of randomized follow-up. However, to achieve these results, sites with a team of advanced cardiologists and nurses dedicated to monitor and support HF outpatients are needed [71]. 

Cardiac contractility modulation (CCM) is an encouraging device treatment for HF patients with an LVEF of 25% to 45% ineligible for cardiac resynchronization therapy, which has shown beneficial effects in improving functional capacity and quality of life by delivering biphasic pulses to the right ventricular septum during the absolute refractory period of the myocardium through one lead in the right atrium and two in the right ventricular septum [72]. These results were shown in the FIX-HF-4 study [73], the FIX-HF-5 trial [74] and the following FIX-HF-5C study [75], which aimed to demonstrate that a two-lead system, without the need of an atrial lead, is equally safe, improving peak oxygen consumption (VO2) and NYHA class with less adverse effect; because the algorithm developed does not require sensing the timing of atrial depolarization, it may be used in patients with atrial fibrillation. 

This implantable electrical therapeutic technology, together with baroreflex activation therapy (BAT) which by increasing parasympathetic activity can reduce peripheral resistance [76], are, however, supported by insufficient evidence for their use to be standardized and more randomized clinical trials are needed.

## 4. Conclusions

HF is a complex and multifaceted syndrome and, currently, there are several pharmacological possibilities to treat it, reducing mortality and rehospitalization rates. There are several gaps both in guidelines and consensus documents regarding the correct initiation and up-titration of HF disease-modifying drugs. For this reason, several authors proposed different approaches mainly based on clinical experience and focused only on chronic stabilized patients, mainly in the outpatient setting. However, the acute decompensation episodes are a critical part of the HF continuum, because they limit GDMT optimization, exposing patients to high rates of mortality and rehospitalization. AHF management is crucial to prepare the field in order to build an optimized therapeutic regimen. Evidence strongly suggests that patients suffering from HF should be treated with a more upfront therapeutic protocol, possibly obtaining a quick hemodynamic stabilization and introducing all the molecules in a short delay and rapidly reaching up-titration, already in the in-hospital setting. This seems to provide both improvement in the quality of life, event free survival and the reduction of preventable hospitalizations and health care expenditure [77]. In conclusion, an approach based on the early use of Levosimendan during the acute phase, in particular when patients are not stable from the hemodynamic point of view, may prepare the field to disease-modifying drugs’ introduction, starting with BB and SGLT2i. The first may be preferred because they may have a great and early impact on arrythmias and death, while SGLT2i may be preferred due to its high tolerability and safety. When hemodynamic stability, as well as blood pressure and renal function stabilization have been reached, ARNI may be introduced before hospital discharge. MRAs, if not necessary during the acute decompensation phase to balance potassium loss induced by diuretics, may be started during follow-up or started earlier at a low dose. Subsequent follow-up visits performed every two weeks should aim to up-titrate GDMT, exploiting potassium binders in patients with CKD and RAASi/MRAs induced hyperkalemia. Moreover, device therapy should be strongly considered in patients still symptomatic despite optimal medical therapy and in patients who do not adequately tolerate disease modifying drugs. 

## Figures and Tables

**Table 1:** Current evidences and indications reported by ESC and ACC/AHA Guidelines regarding the treatment of acute and chronic heart failure.

Pharmacological Treatment of Acute Heart Failure		Pharmacological Treatment of Chronic Heart Failure	
ESC 2021 Guidelines	ACC/AHA 2022 Guidelines	ESC 2021 Guidelines	ACC/AHA 2022 Guidelines
Clinical presentation leads treatment approach: - Acute decompensated HF: Diuretics for decongestion and inotropes for hypoperfusion - Acute pulmonary oedema: Oxygen therapy, i.v. diuretics and i.v. vasodilators to reduce LV afterload, if necessary - Isolated RVF: Diuretics for venous congestion, noradrenaline and/or inotropes for low cardiac output and hemodynamic instability (inotropes reducing cardiac filling pressures may be preferred) - Cardiogenic shock: Oxygen therapy, inotropes/vasopressors, MCS	Treatment approach based on hemodynamic state: - Decongestion strategy: Hospitalized HF patients with fluid overload should be treated with intravenous loop diuretics (to titrate during hospitalization and to adjust before discharge) - Parenteral vasodilation therapy: Vasodilators to relieve pulmonary congestion - Cardiogenic shock: Inotropes, temporary MCS	ACEi/ARNI, BB, MRAs and SGLT2i have been shown to improve survival, reduce the risk of HF hospitalizations in patients with HFrEF. - Up-titration of all disease-modifying drugs to the doses used in the clinical trials (or to maximally tolerated doses if that is not possible). - ARNI may be considered as a first-line therapy instead of an ACEi (de novo patient). - ARNI are recommended as a replacement for ACEi in patients who remain symptomatic on OMT. - SGLT2i reduced the risk of CV death and worsening HF in patients with HFrEF, regardless of diabetes.	ACEi/ARNI, BB, MRAs and SGLT2i have been shown to improve survival, reduce the risk of HF hospitalizations in patients with HFrEF. - Disease-modifying drugs may be started simultaneously at initial (low) doses (ARNI/ACEi/ARB, BB, MRAs, SGLT2i) - Alternatively, they may be started sequentially, basing on clinical factors, without need to achieve target dosing before initiating next medication. - Medication doses should be increased to target as tolerated. - SGLT2i should be considered in patients with HFpEF

ESC: European Society of Cardiology; ACC/AHA: American College of Cardiology/American Heart Association; HF: heart failure; LV: left ventricular; RVF: right ventricular failure; MCS: mechanical circulatory support; ACEi: angiotensin-converting enzyme inhibitors; ARNI: angiotensin receptor neprilysin inhibitors; BB: beta blockers; MRAs: mineralocorticoid receptor antagonists; SGLT2i: sodium glucose cotransporter 2 inhibitors; HFrEF: heart failure with reduced ejection fraction; OMT: optimal medical therapy; CV: cardiovascular; HFpEF: heart failure with preserved ejection fraction.

**Table 2:** Main studies evaluating an approach based on volume status for the management of patients with both acute heart failure and chronic heart failure.

Acute Heart Failure	Main Findings	Chronic Heart Failure	Main Findings
Leahova-Cerchez et al. [21]	Integrated approach based on clinical (JVD, HJR), biological and echocardiographic (IVC) signs of congestion may guide diuretic therapy, reducing the risk of renal failure in patients >75 years old with acute decompensated HF	Khandwalla et al. [24]	Increasing IVC diameter, as demonstrated by ultrasound, is associated with increased risk for HF hospitalization and may be useful to manage patients.
Kobayashi et al. [22]	The estimated PV status at discharge, on top of classical prognostic markers, may improve risk stratification for the composite outcome of rehospitalization due to worsening HF and all-cause mortality in patients admitted due to acute decompensated HF	Miller et al. [25]	Patients with hypervolemia show high filling pressure, but patients with euvolemia may also show high filling pressure. This is mainly determined by the severity of myocardial dysfunction. Integrated approach based on myocardial function, cardiac filling pressure and intravascular volume evaluation is needed for optimal HF management
Van Aelst et al. [23]	In patients with AHF, higher E/e’, larger left and right atria, higher IVC diameter with lower variability and higher pulmonary artery systolic pressure compared with non-cardiac dyspnea have been demonstrated. The biomarkers sCD146 and MR-proANP, but not BNP, were associated with echocardiographic parameters suggestive of venous congestion. The venous congestion state in acute settings is similar between HFrEF and HFpEF, despite HFrEF patients showing higher BNP values	Ling et al. [26]	Relative PV status calculation defines how patients with CHF deviate from their ideal volume status, and it is independently associated with outcomes

JVD: jugular venous distension; HJR: hepatojugular reflux; IVC: inferior vena cava; HF: heart failure; PV: plasma volume; AHF: acute heart failure; MR-proANP: midregional pro-atrial natriuretic peptide; BNP: brain natriuretic peptide; HFrEF: heart failure with reduced ejection fraction; HFpEF: heart failure with preserved ejection fraction; CHF: chronic heart failure.

**Table 3:** Proposed algorithms of guidelines-directed medical therapy initiation and up-titration in patients with heart failure with reduced ejection fraction.

Authors	Strategy of GDMT Up-Titration	Target
McMurray et al. [60]	Upfront initiation of BB and SGLT2i (step 1), followed by ARNI within two weeks (step 2) and MRAs two more weeks later (step 3)	Achievement of GDMT within 4 weeks
Miller et al. [61]	Cluster scheme: Cluster (A) SGLT2i and diuretics for volume overload; Cluster (B) ARNI/MRAs for hypertension and Cluster (C) BB and SNI for high heart rate. Initiation of BB, ACEi/ARNI, MRAs and SGLT2i before single drug up-titration.	Weekly up-titration and achievement of GDMT within 2/3 months
Tomasoni et al. [13]	Early upfront administration of SGLT2i due to safety and tolerability; low dose initiation of BB, ACEi/ARNI and MRAs and subsequent up-titration as tolerated. Sequence of optimization should be based on patient’s characteristics.	Achievement of GDMT within 42 days
Greene et al. [62]	Nearly simultaneous introduction of low doses of each of the four classes of drugs during the first week. Up-titration every two weeks for BB, first up-titration suggested after 4 weeks for ARNI and MRAs	Achievement of GDMT within 42 days. Subsequently consider further up-titration, if possible, or device therapy, if needed.

GDMT: guidelines-directed medical therapy; BB: beta blocker; SGLT2i: sodium-glucose cotransporter 2 inhibitor; ARNI: angiotensin receptor neprilysin inhibitors; MRAs: mineralocorticoid receptor antagonists; SNI: sinus node inhibitors; ACEi: angiotensin-converting enzyme inhibitor.
